# Effect of Modification Rules in Competition on Technical–Tactical Action in Young Tennis Players (Under-10)

**DOI:** 10.3389/fpsyg.2019.02789

**Published:** 2020-01-07

**Authors:** José María Gimenez-Egido, Enrique Ortega-Toro, José M. Palao, Isidro Verdú-Conesa, Gema Torres-Luque

**Affiliations:** ^1^Department of Physical Activity and Sport, Faculty of Sport Science, University of Murcia, Regional Campus of International Excellence “Campus Mare Nostrum”, Murcia, Spain; ^2^Health, Exercise Science, and Sport Management, University of Wisconsin–Parkside, Kenosha, WI, United States; ^3^Department of Computing and Systems, Faculty of Computer Science, University of Murcia, Regional Campus of International Excellence “Campus Mare Nostrum”, Murcia, Spain; ^4^Faculty of Education, University of Jaen, Jaen, Spain

**Keywords:** performance analysis, equipment scaling, young players, small-sided games, singles tennis

## Abstract

Adapting competitions to young players’ characteristics is an important pillar in the optimal teaching–learning process. The objective of the present study is to analyze the effect of modifying net height (from 0.91 to 0.80 m) and court dimensions (from 23.77 × 8.23 m to 18 × 8.23 m) for under-10 (U-10) tennis players on the following: (a) kinds of technical and tactical basic, situational, and special strokes; (b) tennis players’ hitting area; (c) landing location of the serve; (d) ball landing location after the serve; (d) stroke effectiveness; and (e) rally length. The study design was quasi-experimental in nature, observing the fluctuation/change in technical–tactical variables of the tennis players when playing a “Tennis 10s Green Competition” (GC) with the current federative rules and a redesigned competition “Modified Competition” (MC) including altered net height and court dimensions based on small-sided games (SSGs) and equipment scaling. Twenty U-10 tennis players were studied (age of players = 9.46 ± 0.66 years, average weekly training in tennis = 2.90 ± 1.07 h, years of experience = 3.65 ± 1.53 years). The results showed that in MC, there was a greater technical–tactical variability compared with the GC in terms of the following: (a) greater service effectiveness; (b) more situational and special strokes; and (c) a more equitable change in the distribution of hitting and ball landing locations. The values of MC showed that the current adaptation rules and equipment in federated U-10 competitions might not be enough to improve the teaching–learning process under the comprehensive approach. The current competition for U-10 tennis players (stage green) should be redesigned, in order to build an optimal process of affordances to develop a multidimensional positive impact during this training stage.

## Introduction

Competition is a dynamic and complex formative environment if it is accurately conducted at early training stages because of its multidimensional effect on many factors (Schmidhofer et al., 2014; Bayer et al., 2017; Krause et al., 2019). In recent years, there has been an increasing amount of literature about the research topic in the following sports: (a) tennis (Bayer et al., 2017; Fitzpatrick et al., 2017; Limpens et al., 2018); (b) basketball (Ortega et al., 2015); (c) soccer (Casal et al., 2017; Ortega-Toro et al., 2018); (d) handball (García-Angulo et al., 2019); (e) cricket (Elliott et al., 2005; Harwood et al., 2018; Takamido et al., 2019); (f) rugby (Bennett et al., 2016; Morley et al., 2016; Elliott et al., 2019); (g) volleyball (Gillham and Gut, 2012); and (h) flag football (Burton et al., 2011a). Overall, these studies highlight that the need for adapting competition at early training does not provide an integral development of young athletes (Burton et al., 2011b; Ortega et al., 2015; Buszard et al., 2016; Gonçalves et al., 2016; McCarthy et al., 2016). This problem encourages science to seek methodological approaches that will aid coaches, federations, and sports institutions in designing competitions that will improve the teaching–learning processes of young players (Araujo et al., 2006; Davids et al., 2013b; McCarthy et al., 2016; Peráèek and Peráèková, 2018).

To develop competitions that improve the teaching–learning process at the training stages, current competitions are based on small-sided games (SSGs) from a comprehensive approach (Renshaw et al., 2010; Davids et al., 2012, 2013a). The goal of this comprehensive approach is to adapt the competition holistically to young players by modifying the rules of the game, rules of the league, sports equipment, and playing spaces (task constraints) (Buszard et al., 2016; McCarthy et al., 2016; Hastie et al., 2017; Oppici et al., 2017; Ortega-Toro et al., 2018).

One major issue in early tennis research concerned scaling of sport equipment (e.g., balls, rackets, and nets) and playing spaces (e.g., type of surface and court dimensions) to facilitate its practice, which was developed by Haake et al. (2003); Mehta and Pallis (2003), and Cooke and Davey (2005). On the basis of these studies, some associations and federations [e.g., United States Tennis Association’s Project 36/60, Tennis Australia’s MLC Tennis Hot Shots, and International Tennis Federation’s (ITF) Play and Stay Program] have understood the need to adapt the competition to the characteristics of developing players (International Tennis Federation [ITF], 2011, 2013). Perhaps, the greatest exponent is the Play and Stay Program, which established a competition system called “Tennis 10s” divided into three stages: Red (age = 5–8), Orange (age = 8–10), and Green (age = 9–10), scaling court dimensions, type of ball, and size of tennis rackets depending on player age (International Tennis Federation [ITF], 2011, 2013). Conversely, Ebert (2012) argued that the Green stage competition is not adapted to under-10 (U-10) tennis players’ characteristics and proposes a new stage called “Lime,” because of observed low success values in serving and little variability of tennis drills. However, a major problem was to modify both competitions by only focusing on players’ physical aspects (e.g., shorter racquets for shorter players) based on the opinion/experience of coaches, theoretical frameworks, and early studies (Larson and Guggenheimer, 2013; Buszard et al., 2014; Schmidhofer et al., 2014).

In this sense, few studies have objectively analyzed the actual impact of these scaled competitions in tennis (Schmidhofer et al., 2014; Bayer et al., 2017). The research has mostly focused the analyses on two factors: (a) reducing court dimensions and (b) decreasing net height to identify the leading constraints approach in the Green stage (Schmidhofer et al., 2014; Kachel et al., 2015; Buszard et al., 2016; Bayer et al., 2017). However, far too little attention has been paid to analyzing total technical–tactical actions to identify task constraints in competitions holistically (Torres-Luque et al., 2017). As a result, the U-10 tennis stage must be assessed to know if the competition creates affordances for improved optimal learning opportunity (Tan et al., 2012; Harvey and Jarrett, 2014; Gonçalves et al., 2016; Hastie et al., 2017).

The key research question that will be tested is whether Modified Competition (MC) adapting net and court dimensions is likely to generate a greater amount and variability of strokes than current Green Competition (GC). The aim of this study is to investigate the effect of MC by reducing net height (from 0.91 to 0.80 m) and court dimensions (from 23.77 × 8.23 m to 18.00 × 8.23 m), besides observing the differences with the current U-10 tennis players’ competition (GC) in the following technical–tactical aspects: (a) basic, situational, and special technical–tactical kinds of strokes; (b) players’ hitting area; (c) ball landing location of the serve; (d) ball landing location after the serve; (e) ball trajectory after the serve; (f) stroke effectiveness; and (g) rally length.

## Materials and Methods

### Design

The study was a quasi-experimental cross-sectional pretest–posttest design (Ato et al., 2013). To analyze the technical–tactical parameters of both competitions, an observational, nomothetic, multidimensional, and continuous intra-sessional registration study was used (Anguera et al., 2011; Anguera and Hernández-Mendo, 2013). This study respected the ethical principles established by the United Nations Educational, Scientific and Cultural Organization (UNESCO) Declaration on Bioethics and Human Rights. The study was approved for development by the Ethics Committee of the University of Murcia (Spain) (ID 1925/2018). Following the Declaration of Helsinki, the players voluntarily participated in the study, and written informed consent was obtained from and signed by the parents/guardians of all participants for the development of this study.

### Participants

Twenty tennis players (U-10) took part in the study. The sample selection was carried out through an intentional sampling method according to the criteria of accessibility and proximity (the specificity in the study design marked the non-randomized sample) (Otzen and Manterola, 2017). To control internal validity of the sample, the following actions were carried out: (a) a pretest–posttest design (reduction of biases to compare different sample groups); (b) similar samples’ characteristics (age of players = 9.46 ± 0.66 years; dominant hand = 20 right-handed; type of backhand = 20 two-handed backhand; average weekly training in tennis = 2.90 ± 1.07 h; years of experience = 3.65 ± 1.53 years; weight = 34.80 ± 6.59 kg; height = 136.00 ± 7.94 cm; abdominal perimeter = 64.44 ± 7.63 cm; VO_2_max (Course Navette) = 20.90 ± 4.57 ml kg^–1^ min^–1^; manual dynamometry = 14.84 ± 3.33 kg); and (c) a week gap between the pretest and posttest to eliminate bias due to the subjects’ maturation (Thomas and Nelson, 2007). The study sample was the strokes (*n* = 10.056) made by players in both competitions.

To maintain stability between GC and MC, both competitions met the following common features: (a) the same number of total matches (40 matches) were played in each competition; (b) four matches with a short-set format (one set to four games) with a tiebreak of 7 points (in case of a tie) were played by each player; (c) players were randomly assigned to a group of five players; (d) competitions were played with the round-robin system; (e) the average match duration was 23.60 ± 6.08 min; (f) the rest time between matches was at least the duration of a match plus 10 extra minutes (average rest between matches = 33.60 ± 6.03 min) to avoid fatigue (Limpens et al., 2018); and (g) the matches were played in the same order and schedule.

**FIGURE 1:**
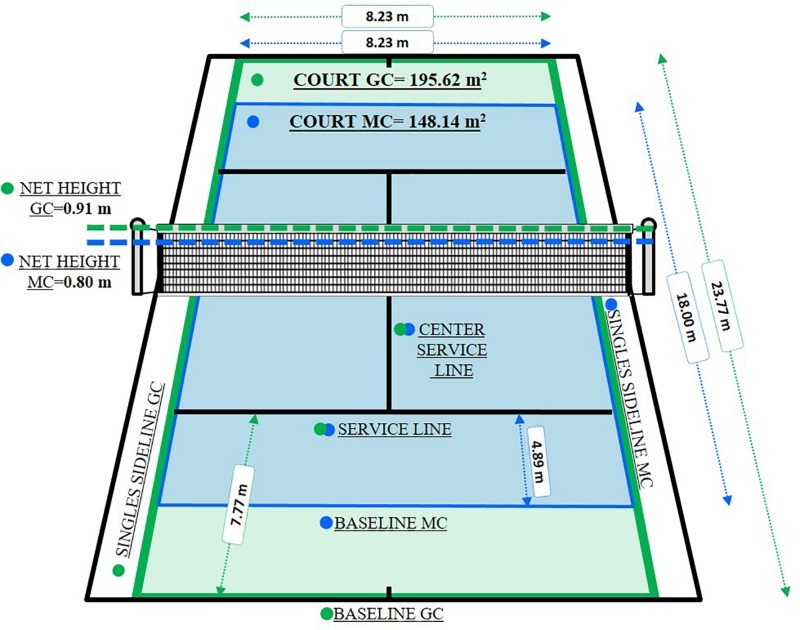
Illustration of net height and court dimensions in both competitions (Tennis 10s Green Competition “GC” = green color and Modified Competition “MC” = blue color).

GC was played according to the ITF rules based on the Tennis 10s Green Competition System (International Tennis Federation [ITF], 2011, 2013) (Figure 1). MC was based on a combination of rules and equipment of the Tennis 10s competition system (net height and court dimensions of the Orange stage and balls of the Green stage), redesigning a new format of competition (Figure 1).

### Instruments

The “Observational Instrument for the Technical–Tactical Actions in Singles Tennis” (Torres-Luque et al., 2018) was used according to the study’s objectives (Anguera et al., 2018a; Chacón-Moscoso et al., 2018).

The instrument was applied to evaluate the player’s stroke across three key criteria: context, result, and game. Three types of criteria were observed; however, the criterion “game” was selected to respond to the research problem. The “game” criterion is composed of five variables: kinds of technical and tactical strokes, ball landing location, player’s hitting area, stroke effectiveness, and rally length (Table 1). To provide useful data to coaches/educators, it was decided to merge some categories of the variable “kinds of technical and tactical stroke.” In this vein, it was decided to merge the categories “degree wide and body area of both sides in the serve” (deuce and advantage) following the recommendations of the Andalusian Tennis Federation’s training manual for young tennis players (Torres-Luque et al., 2010) and the results in Schmidhofer et al. (2014). The main reason was that players, during this formative stage, have a clear intention to serve into the “T” and wide areas, as shown by Schmidhofer et al. (2014). The second reason was the high probability of serving with intentionality of angle and bouncing in the body area, because of the sample’s characteristics and the service trajectory (cross-court) (Torres-Luque et al., 2010).

**TABLE 1:** Macro variables, micro variables, and initial category of the observational instrument (initial category) and their final transformation for this study (final category).

***Macro variable kinds of technical and tactical stroke***	

***Micro variables basic strokes***	

**Initial category**	**Final category**
First service	First service
Second service	Second service
Forehand return	Forehand return
Two-handed backhand return/one-handed backhand return	Backhand return
Forehand groundstroke	Forehand groundstroke
Two-handed backhand groundstroke/one-handed backhand groundstroke	Backhand groundstroke
Forehand volley	Forehand volley
Backhand volley	Backhand volley
Smash	Smash

***Micro variable special strokes***	

Forehand lob/two-handed backhand lob/one-handed backhand lob	Lob stroke
Forehand drop/two-handed backhand drop/one-handed backhand drop	Return stroke
Forehand half volley/two-handed backhand half volley/one-handed backhand half volley	Half volley stroke

***Micro variable situational strokes***	

Forehand lob return/two-handed backhand lob return/one-handed backhand lob return	Lob return stroke
Forehand drop return/two-handed backhand drop return/one-handed backhand drop return	Drop return stroke
Forehand approach/two-handed backhand approach/one-handed backhand approach	Approach stroke
Forehand counter drop/two-handed backhand counter drop/one-handed backhand counter drop	Counter drop stroke
Forehand passing/two-handed backhand passing/one-handed backhand passing	Passing stroke
Two-handed backhand return/one-handed backhand return	Backhand return stroke
Forehand drop return/two-handed backhand drop return/one-handed backhand drop return	Drop return stroke
Forehand return approach/two-handed backhand return approach/one-handed backhand return approach	Return approach stroke
Forehand passing of return/two-handed backhand passing of return/one-handed backhand passing of return	Passing return stroke

***Macro variable player’s hitting area***	

**Initial category**	**Final category**

Behind the baseline in the central area	Behind the baseline in the central area
Behind the baseline in the right area	Behind the baseline in the right area
Behind the baseline in the left area	Behind the baseline in the left area
Inside the court and behind the service line in the central area	Inside the court and behind the service line in the central area
Inside the court and behind the service line in the right area	Inside the court and behind the service line in the right area
Inside the court and behind the service line in the left area	Inside the court and behind the service line in the left area
Between the service line and the net in the central area	Between the service line and the net in the central area
Between the service line and the net in the deuce area	Between the service line and the net in the right area
Between the service line and the net in the advantage area	Between the service line and the net in the left area
Right singles sidelines on the deuce side	Outside of the singles sideline on the deuce side
Left singles sidelines on the advantage side	Outside of the singles sideline on the advantage side

***Macro variables landing location***	

***Micro variables landing location of serve***	

**Initial category**	**Final category**

The wide area of the deuce side/the body area of the deuce side	The wide and body areas of both sides of the deuce side
The wide and body areas of both sides of the advantage side	The wide and body areas of both sides of the advantage side
The T area of the deuce side	The T area of the deuce side
The T area of the advantage side	The T area of the advantage side
Net error	Net error
Out of the service line	Out of the service line
Out of the center service line on the deuce side/out of the center service line on the advantage side	Out of the center service line
Out of the singles sideline on the deuce side	Out of the singles sideline on the deuce side
Out of the singles sideline on the advantage side	Out of the singles sideline on the advantage side
–	Error (ball landing on the player’s hitting side)

***Micro variable ball landing location after the serve***	

The opponent hits the ball without previous bounce	The opponent hits the ball without previous bounce
The central area between the net and the service line	The central area between the net and the service line
The right area between the net and the service line	The right area between the net and the service line
The left area between the net and the service line	The left area between the net and the service line
Inside the court behind the service line in the right area	Inside the court behind the service line in the right area
Inside the court behind the service line in the central area	Inside the court behind the service line in the central area
Inside the court behind the service line in the left area	Inside the court behind the service line in the left area
Net error	Net error
Out of the baseline	Out of the baseline
Out of the singles sideline on the deuce side	Out of the singles sideline on the deuce side
Out of the singles sideline on the advantage side	Out of the singles sideline on the advantage side
–	Error (ball landing on the player’s hitting side)

***Macro variable stroke effectiveness***	

**Initial category**	**Final category**

Ace (*a serve made by a player in which that player gets the point directly, without his/her opponent having touched the ball*)	Ace
Winner (*a stroke made by a player in which that player gets the point directly, without his/her opponent having touched the ball*)	Winner
Transition stroke (*a stroke made by a player after which the opponent hits the ball and it bounces inside the court of the first player*)	Transition stroke
Previous stroke of an opponent error (*a stroke made by a player after which the opponent hits the ball and commits an error, losing the point*)	Previous stroke of an opponent error
Error (*a player hits the ball and sends it out of the regulatory area of the court or to the net point*)	Error
Let (*repeat the serve if the ball touches the net and lands in the opponent’s serve box*)	Let

***Macro variable rally length***	

**Initial category**	**Final category**

1 stroke	1 stroke
From 2 to 5 strokes	From 2 to 5 strokes
From 6 to 9 strokes	From 6 to 9 strokes
10 or more stroke	10 or more stroke

### Procedure

The matches were recorded using two cameras in the background (calibrated at a height of 2.40 m above ground and at a distance of 6.40 m from the baseline). The recordings were analyzed through systematic and direct observation using the “Kinovea-0.8.15” computer software on a double screen and a “perspective grid” tool to delimit the field format. The protocol of continuous recording of all technical–tactical behaviors was performed as recommended by Anguera et al. (2018b).

### Data Quality Control

The collection of data was carried out by two observers possessing a degree in Sciences of Physical Activity and Sports with a specialization in “Racket Sports” and the federative title of “Tennis Coach Level II.” Observer training was conducted according to the training protocols designed previously in several investigations (Losada and Manolov, 2014; Gamonales et al., 2018). The observers performed the following training steps: (a) theoretical training by studying the use and terminology of the observational instrument; (b) practical training with the calculation of intraobserver reliability, recording 20% of behaviors in a match; (c) practical training with the calculation of interobserver reliability, recording 33% of behaviors in another match, with 1 week apart; and (d) calculation of interobserver and intraobserver reliability values, which were found to be in line with other investigations of performance analysis (Gómez et al., 2018; Sainz de Baranda et al., 2019; Torres-Luque et al., 2019) in tennis (Losada et al., 2015; Fitzpatrick et al., 2018). The calculation of intraobserver and interobserver reliability was carried out through the weighted kappa statistic for ball landing locations and player’s hitting areas recommended by Robinson and O’Donoghue (2007). However, Cohen’s kappa was used to analyze intraobserver and interobserver reliability in all other variables. The values of agreement by the two observers were “very good” in all variables (“Agreement of technical–tactical variables coded by independent observers”; see Supplementary Data Sheet 1). To know the values of intra- and inter-reliability, the following intervals were used: <0.20 poor, 0.21–0.40 fair, 0.41–0.60 moderate, 0.61–0.80 good, and 0.81–1.00 very good (Altman, 1991). The statistical program used was the statistical package IBM SPSS Statistics 23.0 (IBM Corp., Armonk, NY, United States).

### Data Notation

Data recording was completed via manual notation into an Excel spreadsheet. Thus, all technical–tactical actions were registered sequentially, each row representing one shot and each column being a different variable of study. Subsequently, exploratory data analysis was done to perform the initial investigation, as well as to discover patterns and detect anomalies with summary statistics (Field, 2013). Finally, the number of technical–tactical actions (columns) performed by young tennis players per match was counted (rows) for further statistical analysis (analysis of variance).

### Statistical Analysis

The statistical analysis was divided into three phases: (a) descriptive analysis; (b) analysis of variance; and (c) effect size analysis. In the first phase related to the descriptive analysis, the mean values, percentages, and standard deviation of each analyzed category were calculated. The second phase of analysis was based on an unconditional analysis model using Student’s *t*-test for paired samples, establishing statistical difference in *p* < 0.05 (Bono Cabre, 2012). The third phase was the calculation of the effect size according to the recommendations provided by the American Psychological Association (2010). The effect size was calculated through Cohen’s *d* (1988), considering the values as a small effect (*d* < 0.2), medium effect (0.2 ≤ *d* < 0.6), high effect (0.6 ≤ *d* < 1.2), and strong effect (*d* > 1.2). Interpretation of the effect size will be carried out from a twofold perspective: (a) extrapolation of effect size values to the sample and (b) magnitude of the differences found (Coe and Merino, 2003). From another perspective, confidence intervals (CIs) were calculated to contribute to the development of science for this study to be taken into account as a sample for a future meta-analysis (Cárdenas and Arancibia, 2014). The effect size and the analysis of variance were obtained through the spreadsheet jamovi 1.1.5 based on the graphical user interface R.

## Results

**FIGURE 2:**
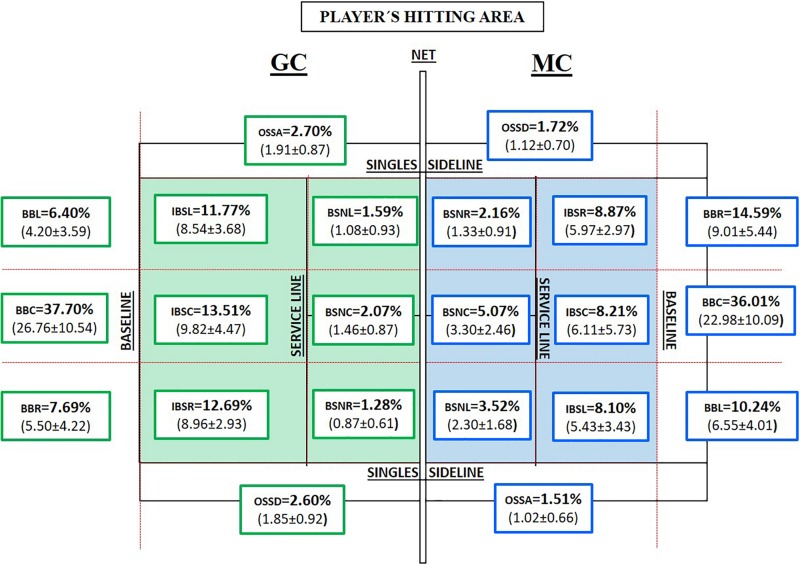
Percentage and mean of the tennis players’ hitting area per match in both competitions (Tennis 10s Green Competition “GC” = green color and Modified Competition “MC” = blue color). BBR, behind the baseline in the right area; BBC, behind the baseline in the central area; BBL, behind the baseline in the left area; IBSR, inside the court behind the service line in the right area; IBSC, inside the court behind the service line in the central area; IBSL, inside the court behind the service line in the left area; BSNR, between the service line and the net right area; BSNC, between the service line and the net central area; BSNL, between the service line and the net left area; OSSD, out from the singles sideline on the deuce side; OSSA, out from the singles sideline on the advantage side.

**FIGURE 3:**
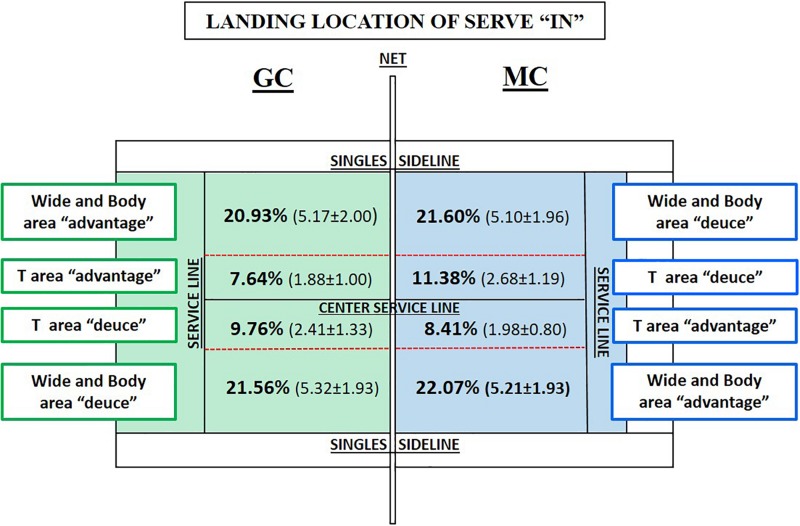
Percentage and mean of the landing locations of the serve “in” per match in both competitions (Tennis 10s Green Competition “GC” = green color and Modified Competition “MC” = blue color).

**FIGURE 4:**
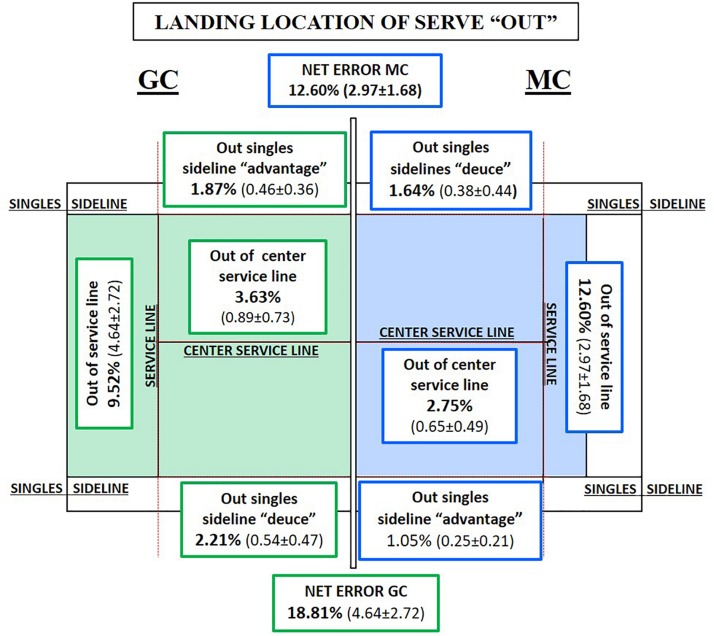
Percentage and mean per match of the landing locations of the serve “out” per match in both competitions (Tennis 10s Green Competition “GC” = green color and Modified Competition “MC” = blue color).

**FIGURE 5:**
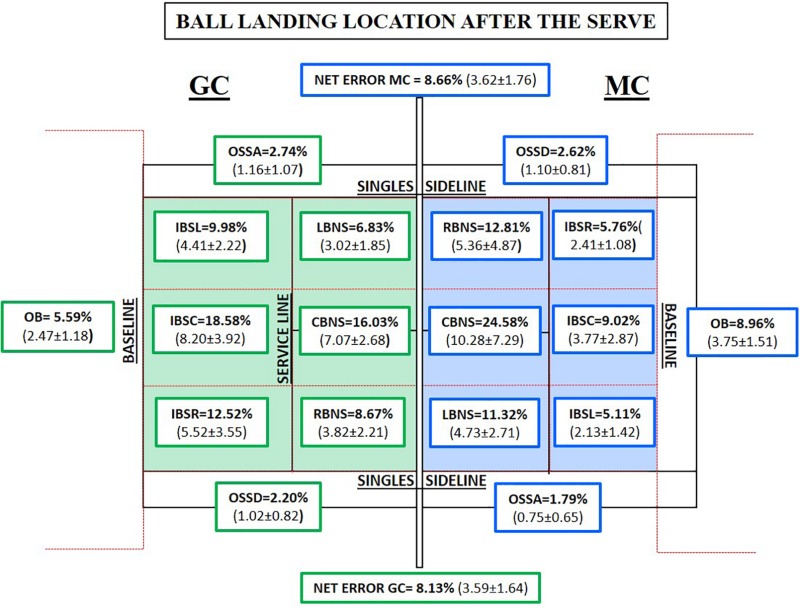
Percentage and mean per match of ball landing locations after the serve per match in both competitions (Tennis 10s Green Competition “GC” = green color and Modified Competition “MC” = blue color). IBSR, inside the court behind the service line in the right area; IBSC, inside the court behind the service line in the central area; IBSL, inside the court behind the service line in the right area; RBNS, the right area between the net and the service line; CBNS, the central area between the net and the service line; LBNS, the left area between the net and the service line; OSSD, out from the singles sideline on the deuce side; OSSA, out from the singles sideline on the advantage side.

Results corresponding to the technical–tactical performance analysis in different variables can be observed in Tables 2–5 and Figures 2–5.

**TABLE 2:** Descriptive values (mean, standard deviation, and percentage) and analysis of variance (Student’s *t*-test for paired samples) of the “kinds of basic strokes” per match.

**Kinds of basic strokes**	**GC**		**MC**		**Confidence interval**		***Post hoc***	**Effect size**
					
	**$\bar{X}$/*S***	**%**	**$\bar{X}$/*S***	**%**	**Lower**	**Upper**	***p* < 0.05**	***d***
First serve	16.80 ± 5.62	25.79	15.84 ± 5.06	26.66	–3.179	1.241	0.370	0.205
Second serve	8.05 ± 2.56	13.29	6.34 ± 2.64	11.53	0.289	3.127	0.021^∗^	0.563
Forehand return	9.65 ± 4.26	14.55	8.70 ± 4.02	14.01	–2.571	0.66294	0.232	0.276
Backhand return	3.39 ± 2.09	5.03	3.72 ± 2.06	6.57	–1.196	0.537	0.436	0.177
Forehand	16.05 ± 8.43	22.83	13.61 ± 12.14	19.14	–3.151	8.021	0.373	0.204
Backhand	7.86 ± 5.62	10.74	6.26 ± 4.56	9.52	–4.692	1.492	0.292	0.242
Forehand volley	0.91 ± 0.93	1.25	0.70 ± 0.79	1.15	–0.250	0.677	0.347	0.215
Backhand volley	0.50 ± 0.66	0.74	0.42 ± 0.50	0.72	–0.323	0.173	0.535	0.141
Smash	0.40 ± 0.45	0.59	0.23 ± 0.39	0.38	–0.107	0.173	0.222	0.282
Others	0.14 ± 0.26	0.17	0.14 ± 0.21	0.40	–0.143	0.145	0.989	0.003
Total	63.80 ± 22.83	100	56.02 ± 22.49	100	–2.60	18.18	0.133	0.351

*GC = Tennis 10s Green Competition, Modified Competition = MC. $\bar{X}$ = mean, *S* = standard deviation. *Post hoc*: statistically significant differences = *p* < 0.05^∗^. Effect size = Cohen’s *d*.*

### Kinds of Technical and Tactical Stroke

The average, percentage, and standard deviation of the different kinds of strokes per match can be seen in Tables 2 (basic strokes) and 3 (special and situational strokes).

**TABLE 3:** Descriptive values (mean, standard deviation, and percentage) and analysis of variance (Student’s *t*-test for paired samples) of the “situational and special strokes” per match.

**Kinds of situational and special strokes**	**GC**		**MC**		**Confidence interval**		***Post hoc***	**Effect size**
					
	**$\bar{X}$/*S***	**%**	**$\bar{X}$/*S***	**%**	**Lower**	**Upper**	***p* < 0.05**	***d***
Passing return	0.00 ± 0.00	0.00	0.50 ± 0.10	0.10	0.001	0.098	0.042^∗^	0.487
Lob return	0.05 ± 0.11	0.12	0.60 ± 0.52	1.02	−0.790	–0.308	< 0.001^***^	1.069
Drop return	0.52 ± 0.77	0.77	0.51 ± 0.62	0.82	−0.486	–0.461	0.956	0.0012
Approach	0.08 ± 0.23	0.09	0.83 ± 0.69	1.44	−1.081	–0.435	< 0.001^***^	1.100
Passing	0.02 ± 0.07	0.04	0.26 ± 0.38	0.46	0.049	0.425	0.016^∗^	0.591
Lob	2.10 ± 1.95	2.69	1.88 ± 2.06	2.58	−0.960	1.393	0.705	0.086
Drop	0.76 ± 0.94	1.13	1.17 ± 1.15	1.74	−0.055	0.874	0.081	0.412
Counter drop	0.01 ± 0.07	0.15	0.11 ± 0.20	0.16	−0.010	0.203	0.075	0.421
Half volley	0.07 ± 0.18	0.10	1.08 ± 1.12	1.51	0.479	1.553	< 0.001^***^	0.886
Total	3.64 ± 2.74	100	6.53 ± 4.18	100	−4.570	–1.200	0.002^∗∗^	0.803

*GC = Tennis 10s Green Competition, Modified Competition = MC. $\bar{X}$ = mean, *S* = standard deviation. *Post hoc*: statistically significant differences (*p* < 0.05^∗^, *p* < 0.01^∗∗^, *p* < 0.001^∗∗∗^). Effect size = Cohen’s *d*.*

The values found in terms of the total basic strokes made in each competition show great similarity between both competitions (Table 2), not appreciating statistically significant differences between GC and MC [*t* = 1.570, *df* = 19, mean difference (MD) = 7.79]. However, the category “second serve” showed significant differences with approximately two more services per match being made in the GC with respect to the MC (*t* = **−**2.520, *df* = 19, MD = 1.708). Specifically, CM has a probability of getting between 69 and 73% of lower average values regarding GC (medium effect). There was a shortage of net strokes (smash, forehand, and backhand volleys) in both competitions, with percentage values that do not reach 1.00%. In another sense, there was a clear predominance of the use of forehand over backhand, doubling its value in both competitions.

However, the values of “special and situational strokes” collected (Table 3) indicated a change in the previous trend. Statistically significant differences were observed in the total of special and situational strokes (*t* = 3.590, *df* = 19, MD = 2.89) with a high effect size in favor of MC (approximately 79% of the average values are lower in GC). The category “passing and lob return” presented significant differences in favor of MC (passing return: *t* = 2.179, *df* = 19, MD = 0.05; lob return: *t* = 4.78, *df* = 19, MD = 0.549). The lob return obtains a probability of approximately 84%, with average values that are lower in GC (high effect). Continuing with the previous observation, three of the six categories corresponding to “special strokes” got statistically significant differences in support of MC (approach: *t* = 4.912, *df* = 19, MD = 0.785; passing: *t* = 2.646, *df* = 19, MD = 0.237; half volley: *t* = 4.794, *df* = 19, MD = 0.213). The GC shows a probability of obtaining lower mean values in (a) passing (69–73%, medium effect); (b) half volley (79–82%, high effect); and (c) approach (84–88%, high effect) (Table 3).

To obtain a visual perspective of the hitting areas where the players impacted the ball during the matches, Figure 2 shows in MC a higher percentage of hits being made behind the baseline (60.84%) than those being made in GC (51.78%), although no statistically significant differences were observed. MC evidenced better values in areas near the net (10.75%) compared with GC (4.95%) with statistically significant differences (*p* < 0.001, *t* = 4.057, *df* = 19, MD = 3.63). On the other hand, MC showed a lower percentage of hitting inside the court and behind the service line (25.18%) than that shown in GC (37.97%), with statistically significant differences (*p* < 0.05, *t* = 3.136, *df* = 19, MD = 9.122). Likewise, it can be seen that in MC, the percentage of hits in the right and left court areas (51.70%) is higher than that in GC (41.41%). Although in GC, the strokes from the center of the court (53.28%) surpass those in MC (49.29%), there were no statistically significant differences. In relation to effect size analysis, GC shows a probability of obtaining lower mean values with respect to MC in areas close to the net, approximately 82% of the sample (*d* = 0.907, high effect); in contrast, GC shows the highest average values of hitting behind the service line inside the court (76% of the sample, *d* = 0.700, high effect).

### Landing Location of the Serve

In the analysis of the landing locations of the serve “in” (Figure 3) and “out” (Figure 4), it can be seen that 63.84% of serves, in MC, bounce “in,” while in GC, that number is 59.91% (*p* < 0.001, *t* = 20.907, *df* = 19, MD = 22.295). The percentage of serves “out” in MC is lower (36.40%) than that in GC (39.54%) (*p* < 0.001, *t* = 5.519, *df* = 19, MD = **−**6.758). The area of error serves was 5.90% in MC and 4.07% in GC.

In relation to “landing locations” of the serve, three categories showed significant differences in favor of MC (“net error”: *p* = 0.026, *t* = 2.420, *df* = 19, MD = 1.67; “out of the center service line”: *p* = 0.045, *t* = **−**2.144, *df* = 19, MD = **−**0.245; and “out of the singles sidelines on the advantage side”: *p* ≤ 0.034, *t* = **−**2.280, *df* = 19, MD = **−**0.212). The effect size analysis provides probability for average values of GC to be lower in the following way: (a) 99.9% “bounce inside the service box” (*d* = 4.675, strong effect) and (b) 69% “out of the center service line” (*d* = 0.479, medium effect). In another sense, the likelihood of finding average values lower in MC than in GC is as follows: (a) 88% “bounce out of the service box” (*d* = 1.234, strong effect); (b) 69% “out of singles sidelines on the advantage side” (*d* = 0.510, medium effect); (c) 66% “bounce before the net” (*d* = 0.415, medium effect); (d) 66–69% “out of the service line” (*d* = 0.452, medium effect); and (e) 69–73% “net error” (*d* = 0.541, medium effect).

### Ball Landing Location After the Serve

In the analysis of the ball landing locations after the serve (Figure 5), it can be observed that balls landing in the player’s hitting area (error) have not been included, but their values are 5.36% (GC) and 6.34% (MC). From a depth perspective, it can be seen that in MC, 48.71% of the bounces were produced between the service line and the net; however, in GC, this value was just 31.53% (*p* = 0.016, *t* = **−**2.632, *df* = 19, MD = 6.590). In MC (19.89%), there was a lower percentage of bounces behind the service line to the baseline with respect to GC (41.08%), with statistically significant differences (*p* < 0.001, *t* = 4.560, *df* = 19, MD = 9.472). In the study of the laterality in GC, higher values of ball bounce are seen in lateral areas (38.00%) with respect to MC (35.00%). In addition, the ball bounces 4.38% more in the right side than in the left side in GC; this difference is lower in MC (2.14%). Nevertheless, about ball landing in central areas, the percentage of both competitions reflects a difference of 1%. In particular, ball landing location after the serve shows statistically significant differences in three categories of MC (“out of the baseline”: *p* < 0.001, *t* = **−**3.963, *df* = 19, MD = **−**0.033; the “right area between the net and the service line”: *p* = 0.047, *t* = 2.128, *df* = 19, MD = 3.208; and the “left area between the net and the service line”: *p* = 0.008, *t* = 2.966, *df* = 19, MD = 1.720). In another sense, significant differences were observed in support of GC including the following categories: “inside the court behind the service line in the right area” (*p* < 0.001, *t* = **−**3.914, *df* = 19, MD = **−**3.116), “inside the court behind the service line in the central area” (*p* < 0.001, *t* = **−**4.427, *df* = 19, MD = **−**4.429), and “inside the court behind the service line in the left area” (*p* < 0.001, *t* = **−**4.421, *df* = 19, MD = **−**2.270).

Effect size indicated that the likelihood of finding lower average values in GC is higher than that in MC in the following categories: (a) 82–84% “left area between the service line and the net” (*d* = 0.959, high effect); (b) 79–82% “out of the baseline” (*d* = 0.886, high effect); (c) 66–69% the “right area between the net and the service line” (*d* = 0.475, medium effect); and (d) 79–82% the “central area between the net and the service line” (*d* = 0.875, high effect). However, mean values from MC are lower in comparison with GC: (a) 84% “inside the court behind service line in the central area” (*d* = 0.1.019, high effect); (b) 84% “inside the court behind service line in the left area” (*d* = 0.990, high effect); and (c) 82–84% “inside the court behind service line in the right area” (*d* = 0.959, high effect).

### Stroke Effectiveness

The values presented in Table 4 highlight great similarities between both competitions. However, when observing the category “winner,” there are statistically significant differences to support GC (*t* = −0.879, df = 19, MD = −0.129), with a lower average value between 66 and 69% in MC (ES = medium effect). The category “error” was observed higher than 30% in both competitions.

**TABLE 4:** Descriptive values (mean, standard deviation, and percentage) and analysis of variance (Student’s *t*-test for paired samples) of the “stroke effectiveness” per match.

**Stroke effectiveness**	**GC**		**MC**		**Confidence interval**		***Post hoc***	**Effect size**
					
	**$\bar{X}$/*S***	**%**	**$\bar{X}$/*S***	**%**	**Lower**	**Upper**	***p* < 0.05**	***d***
Ace	0.29 ± 0.46	0.50	0.42 ± 0.51	0.70	−0.178	0.438	0.390	0.196
Winner	3.44 ± 1.98	4.73	2.55 ± 1.40	4.30	0.032	1.751	0.043^∗^	0.485
Transition stroke	34.84 ± 14.24	47.27	30.63 ± 16.69	44.10	−13.71	5.294	0.366	0.207
Previous stroke of an opponent error	10.64 ± 3.37	15.11	11.45 ± 2.76	18.41	−2.087	0.472	0.202	0.295
Error	21.78 ± 4.88	32.18	20.17 ± 5.85	32.04	−3.926	0.710	0.163	0.324
Let	0.15 ± 0.45	0.25	0.26 ± 0.26	0.43	−0.365	0.141	0.364	0.207

*GC = Tennis 10s Green Competition, Modified Competition = MC. $\bar{X}$ = mean, *S* = standard deviation *Post hoc*: statistically significant differences = *p* < 0.05^∗^. Effect size = Cohen’s *d*.*

### Rally Length

The results shown in Table 5 maintain the line of equality between competitions in the variable “rally length.” Just category “1 stroke” showed significant differences in favor of GC (*t* = −2.485, df = 19, MD = −0.991). Average values in the category “1 stroke” (69–73%) per match are lower MC (medium effect).

**TABLE 5:** Descriptive values (mean, standard deviation, and percentage) and analysis of variance (Student’s *t*-test for paired samples) of the “length rally” per match.

**Rally length**	**GC**		**MC**		**Confidence interval**		***Post hoc***	**Effect size**
					
	**$\bar{X}$/*S***	**%**	**$\bar{X}$/*S***	**%**	**Lower**	**Upper**	***p* < 0.05**	***d***
1 stroke	2.90 ± 1.53	20.96	1.91 ± 1.41	14.02	**−**1.826	**−**0.157	0.022^∗^	0.555
From 2 to 5 strokes	10.71 ± 4.75	63.09	11.08 ± 4.26	69.48	**−**1.788	1.052	0.594	0.121
From 6 to 9 strokes	2.06 ± 1.25	11.71	2.04 ± 1.26	13.02	**−**0.715	0.670	0.947	0.0152
10 or more stroke	0.70 ± 0.65	4.22	0.61 ± 0.71	3.46	**−**0.354	0.537	0.672	0.096

*GC = Tennis 10s Green Competition, Modified Competition = MC. $\bar{X}$ = mean, *S* = standard deviation. *Post hoc*: statistically significant differences = *p* < 0.05^∗^. Effect size = Cohen’s *d*.*

## Discussion

This study investigated the impact of decreasing net height and altering court dimensions on U-10 tennis players’ technical and tactical actions. The results show a high equality in terms of the amount and effectiveness of shots between MC and GC. MC shows greater variability and opportunity to practice different strokes and play-patterns, encouraging creative behavior through the experience of various situations.

The lack of learning opportunity and success were clearly observed when analyzing the serve in GC, particularly when comparing the number of the second serves between competitions (number of second serve is lower in MC). Some studies indicate that one of the most important constraint stroke in the teaching–learning process is the serve on the following formative stages: (a) Red stage (Fitzpatrick et al., 2017); and (b) Green stage (Schmidhofer et al., 2014; Timmerman et al., 2015; Bayer et al., 2017; Fitzpatrick et al., 2017; Limpens et al., 2018), Another finding in MC was that serves success improves when net height and court dimensions decreasing, these results match with other studies (Bayer et al., 2017; Limpens et al., 2018). In this line, the analysis of landing locations of serve in both competitions does not show significant differences, furthermore was observed no preferences between landing locations of serve. The tactical intentionality serving is reduced to starting the point, not being used as a technical–tactical action that facilitates winning the point for several reasons: (a) the maturational age of the players is a constraint in the teaching–learning process (Fitzpatrick et al., 2017); (b) lack of SSG experience encourages players to repeat their serve patterns instead of testing new possibilities (Hizan et al., 2015); and (c) the coaches focus their attention mainly on improving ground strokes, spending less time designing tasks to develop the serve. Coaches and federation should address the scarce intentionality/variability in serve directionality at these ages for redesigning competitions and tasks to facilitate serve improvement, as it is an indicator of performance in formative and adult stage (Martin et al., 2018; Fortes et al., 2019). Therefore, net and the court dimensions adaptation seem to facilitate the serves success. In addition, MC gets more errors out of the serviceline; the lack of experience in reduced court enables this result. Meanwhile, in GC, the main error in the serve is the impact of the ball against the net, which is the first obstacle to start the point. These values affirm three needs: (a) modifying the net and the court dimensions to develop children’s tennis to be as similar as possible to that of adults (Torres-Luque et al., 2011; Schmidhofer et al., 2014); (b) adapting the system of competition to the needs and preferences of young tennis players (Ortega et al., 2017; Ortega-Toro et al., 2018); and (c) carrying out longitudinal studies for players to gain experience in SSGs to observe the real impact in play patterns (McCarthy et al., 2016).

In the analysis of the ground strokes in GC and MC, there were more forehand strokes executed than backhand; however, other researchers found that reducing court dimensions promoted the use of backhand (Farrow and Reid, 2010; Fitzpatrick et al., 2017). A possible explanation for these results may be the lack of security when young tennis players hit the ball on the non-dominant side (Reid et al., 2016), which does not allow observing of the real impact of the MC on the use of backhand strokes.

The lack of technical–tactical actions close to the net (volleys and smash) in both competitions is noteworthy, which shows a lack of training using these technical–tactical resources. However, there are studies with the best children’s tennis players in which no volleys were observed in MC than in GC, because of the reduction of net height and court dimensions (Timmerman et al., 2015; Bayer et al., 2017; Fitzpatrick et al., 2017). The main explanations in this study for the technical–tactical actions scarcity near the net could be the lack of experience, lack of training, or even insecurity in volleys, although there was a greater opportunity to hit the ball in areas close to the net in MC (Figure 2).

The shots related to creativity and variability behaviors (situational and special strokes) appear more often in MC. MC seems to encourage the concept of affordance with problem solving through creativity and the theory of “repetition without repetition” that is promoted in the educational context (Hastie et al., 2017; Farias et al., 2019) and in other sports (Torrens et al., 2015). These results should be interpreted with caution because the total percentage of situational and special strokes is not very high (10.45%). It is important to say that intervention time was not enough to develop more creative behaviors, alternative patterns of play, and different types of shots in MC (Timmerman et al., 2015; McCarthy et al., 2016).

By analyzing the fluctuation in “player’s hitting area,” MC shows a greater number of shots close to the net in more offensive areas (inside the service box), improving offensive skills, as advised in several comprehensive methodologies (Burton et al., 2011b; McCarthy et al., 2016; Correia et al., 2019). The distance reduction between the baseline and the net can explain this behavior besides the reduction in net height (Timmerman et al., 2015; Limpens et al., 2018). Another result shows that the predisposition of playing inside the court can be because the court size is too large, not adapting to the children’s characteristics in GC, as shown by Bayer et al. (2017) and Ebert (2012).

When the “landing locations after the serve” were analyzed, it is observed that most bounces are produced between the net and the service line (right and left area) in MC, while in GC as well as in similar studies, most bounces are produced in depth (Timmerman et al., 2015). Regarding the previous idea, this could have a twofold cause. The first could be that in MC, the player plays opening angles not exaggerating topspin, finding patterns of play similar to the adult stage (Bayer et al., 2017; Limpens et al., 2018). Meanwhile, in GC, the player seeks to overwhelm opponents by keeping them away from the baseline with a high bounce to “overcome the net, taking advantage of the court,” so that the adversaries do not hit below the shoulder easily (Farrow and Reid, 2010; Kachel et al., 2015; Timmerman et al., 2015; Fitzpatrick et al., 2017; Limpens et al., 2018). The second proposed cause is the reduction of the court dimensions; this increases the likelihood that the bounce occurs in the service box in MC, while in GC, there is more space between the service line and the baseline. Finally, the increase of the error “out of the baseline” in the MC can be explained because normally the players hit the ball as hard as they do in the regular competition (Green stage).

One factor with a high impact on technical–tactical actions is the “stroke effectiveness” because of its influence on performance and the decision-making process (Farias et al., 2019). In the present study, there can be seen a great equality in the stroke effectiveness between competitions. In contrast, the only category that shows remarkable values in GC compared with MC is the action of winning a point directly (winner). However, previous studies showed that the SSGs improve the accomplishment of winner strokes because of the predisposition to attack (Timmerman et al., 2015; Torres-Luque et al., 2017). Despite this disagreement, both competitions show values far from those found in professional tennis players (Martínez-Gallego et al., 2013). A possible account for this equality in stroke effectiveness can be explained by the lack of previous experiences in MC; future longitudinal studies should check its real impact (McCarthy et al., 2016).

The trend in formative stages is to improve the duration of ball exchange (rally length) to generate learning opportunities in relation to quantity (Farrow and Reid, 2010; Larson and Guggenheimer, 2013; Fitzpatrick et al., 2017). However, the study conducted by Schmidhofer et al. (2014) concluded that an increase in the rally length supported defensive play. Therefore, MC maintains similar rally length values to GC, also improving variability by using more situational and special strokes.

The results suggest that reducing the height of the net and the size of the court in competition creates affordances to optimize teaching–learning processes in U-10 tennis players. This adaptation especially affects the following factors in the teaching–learning processes: (a) improving success in serving; (b) promoting more opportunities to hit the ball in offensive areas (near the net); and (c) enhancing the concept of “repetition without repetition” through more fair distribution of hitting and ball landing locations; and (d) using more situational and special strokes increasing shots’ variability and technical–tactical creative aspects. The main contaminating factor in this study may be the lack of experience of the players in SSGs.

## Conclusion

Net height and court dimension decrease generate a different playing pattern that in GC promotes learning opportunity dependent on quantity and variability of practice. Regarding technical–tactical behavior, MC seems to develop creative strokes and the directionality of the ball in the rally without diminishing other behaviors. In a global sense, GC must be studied extensively, as it is too advanced a stage to meet the needs and preferences of young players (Farrow and Reid, 2010; Schmidhofer et al., 2014; Bayer et al., 2017). Further research might explore which technical–tactical ratios would be ideal at the formative stages for an integral development, whether the competition should create a technical–tactical pattern similar to that used by Association of Tennis Professionals (ATP)/Women’s Tennis Association (WTA) players (Torres-Luque et al., 2011; Schmidhofer et al., 2014; Timmerman et al., 2015), or the development of specific technical–tactical ratios for young tennis players (Timmerman et al., 2015; Gonçalves et al., 2016). Finally, longitudinal studies with different samples’ characteristics playing in different competition formats are required to discover more scientific evidence in this topic (Gonçalves et al., 2016; Fitzpatrick et al., 2018).

## Practical Applications

This research can help to adapt children’s tennis in the following aspects: (a) redesigning sports equipment with greater functionality to meet the need of different formative stages; (b) designing a new competition step between Orange and Green stages; and (c) creating new competition formats in which U-10 tennis players can participate the same day in different match formats. The competition format “Lime” by Ebert (2012), which entails a competition involving court dimensions between the Orange and Green stages, seems the most appropriate option for U-10 elite players; nevertheless, its effect on different samples should be studied. To conclude, the most interesting option under a comprehensive approach to promote learning opportunity would be to create a competition with different match formats (e.g., a Lime court with a net height of 0.75 m, an Orange court with a net height of 0.85 m, and so on) in which the children play at least two or three matches in different competition formats (Chow et al., 2006; Araújo et al., 2015; Ortega and Giménez, 2016; Hastie et al., 2017).

## Data Availability Statement

The datasets generated for this study are available on request to the corresponding author.

## Ethics Statement

The studies involving human participants were reviewed and approved by the University of Murcia. Written informed consent to participate in this study was provided by the participants’ legal guardian/next of kin.

## Author Contributions

JG-E participated in the study design, visualization, data collection, statistical analysis, data interpretation, writing draft preparation, and revision of the manuscript. EO-T participated in the conceptualization, study design, statistical analysis, data interpretation, writing draft preparation, and revision of the manuscript. JP participated in the conceptualization, study design, writing draft preparation, and revision of the manuscript. IV-C participated in the study design, visualization, data collection, and revision of the manuscript. GT-L participated in the conceptualization, study design, data interpretation, writing draft preparation, and revision of the manuscript.

## Conflict of Interest

The authors declare that the research was conducted in the absence of any commercial or financial relationships that could be construed as a potential conflict of interest.
