# Supplementary material for: Effect of Modification Rules in Competition on Technical–Tactical Action in Young Tennis Players (Under-10)
Source: Front Psychol. 2020 Jan 7;10:2789. doi: 10.3389/fpsyg.2019.02789 (PMC6957469; doi:10.3389/fpsyg.2019.02789)
Supplement: Supplementary file 1 [file Data_Sheet_1.docx]

**SUPPLEMENTARY MATERIAL**

| **Supplementary Table 1.**  Agreement of technical-tactical variables coded by independent observers | | | | | | |
| --- | --- | --- | --- | --- | --- | --- |
| **Macro-Variables** | **Intra-reliability observer 1** | | **Intra-reliability observer 2** | | **Inter-reliability** | |
|  | **Kappa Cohen** | **Kappa Weighted** | **Kappa Cohen** | **Kappa Weighted** | **Kappa Cohen** | **Kappa Weighted** |
| Kind of technical and tactical stroke | 0.900 | - | 0.950 | - | 0.950 | - |
| Player´s hitting area | - | 0.911 | - | 0.903 | - | 0.906 |
| Ball landing location | - | 0.992 | - | 0.975 | - | 0.966 |
| Stroke effectiveness | 1.000 | - | 1.000 | - | 0.877 | - |
| Rally length | 0.861 | - | 0.866 | - | 0.820 | - |
